# Association of self-perceived income status with psychological distress and subjective well-being: a cross-sectional study among older adults in India

**DOI:** 10.1186/s40359-021-00588-5

**Published:** 2021-05-18

**Authors:** T. Muhammad, Shobhit Srivastava, T. V. Sekher

**Affiliations:** 1 Department of Population Policies and Programs, International Institute for Population Sciences, Mumbai, Maharashtra India 400088; 2 Department of Mathematical Demography & Statistics, International Institute for Population Sciences, Mumbai, Maharashtra India 400088

**Keywords:** Self-perceived income, Psychological distress, Subjective well-being, Older adults, India

## Abstract

**Background:**

As the older population aged 65 and over worldwide, is estimated to increase from 9% in 2019 to 16% in 2050, rapid aging will transform the aspects such as economic security, employment status, and family structure. The effects of lower levels of perceived income and poor socioeconomic status on the mental health of older adults appear to be large and enduring. Therefore, the present study contributes to the literature on understanding the association of socioeconomic conditions and self-perceived income status in particular, with self-assessed mental health outcomes (psychological distress and subjective well-being) among older adults in India.

**Methods:**

Data for the present study was derived from the Building Knowledge Base on Population Ageing (BKPAI) in India. Bivariate and binary logistic regression analyses were conducted to understand the relationship between socioeconomic status and outcome variables.

**Results:**

About 43% of older adults had no income whereas 7% had income but perceived as not sufficient to fulfil their basic needs. Nearly, 9% of older adults were retired from regular employment. Almost 70% older adults had received no pension and nearly 18% of older adults had no asset ownership. It is revealed that older adults with income that is partially sufficient to fulfil their basic needs were 2.23 times [OR: 2.23, CI: 1.75–2.84] and 1.96 times [OR: 1.96, CI: 1.55–2.47] significantly more likely to suffer from psychological distress and low subjective well-being than those who had income which was sufficient to fulfil their basic needs.

**Conclusions:**

By focusing on four target areas such as the income support, education, family oriented initiatives and local or regional policies, the current framework for assessing the mental health among older adults in India can be modified. A move towards a guaranteed pension for eligible older individuals by which they do not have to remain as a financial burden on their children, may reduce their self-perceived economic distress and result in higher levels of wellbeing in older ages. Also, strategies to address socioeconomic disadvantages and gender differentials related to mental health status among older population are urgently needed.

**Supplementary Information:**

The online version contains supplementary material available at 10.1186/s40359-021-00588-5.

## Background

As the older population aged 65 and over world-wide, is estimated to increase from 9% in 2019 to 16% in 2050, rapid aging will transform the aspects such as economic security, employment status and family structures [1]. Also, India’s older adult population is expected to reach 19% of the total population [2], which in turn, poses a serious challenge to the available geriatric services, including those for mental health. Currently, older individuals who are materially advantaged by their income, education, employment and other resources live a happier and dignified life than those with fewer material resources [3]. Further, socioeconomic status (SES) contributes to subjective well-being (SWB) that reflects life satisfaction and happiness through different mechanisms of individual and aggregate level resources [4]. SES also influences the social and psychological state of older adults and thereby indirectly affects the quality of life in older ages [5].

Various indices of economic hardships such as unemployment, financial strain, and work-related stress, are linked with physical and mental health problems [6–8]. Income and occupational status are independently associated with mental health in most studies in both developed and developing countries, and the effects of low income and poor economic status appear to be large and enduring [9, 10]. However, the experiences of mental health problems and low subjective wellbeing and their disparities across different socioeconomic groups were found to be higher among older population in low- and middle-income countries [11–13]. The economic insecurity of older adults in those countries results in losing their relevance and significance in their own households and increasing feelings of loneliness [14]. Studies have also shown that higher financial strain was associated with more depressive symptoms in older ages [15–17]. Besides, gender, educational level, income, rural residence, and the presence of one or more major medical conditions were associated with increased risk of geriatric depression [18].

In addition, older adults receive support from their multiple social roles such as being a man, being currently married, and having children upon which most interpersonal relationships are based on and the differences in these roles can have a greater impact on their psychological well-being [19]. Furthermore, a growing body of research shows that unlike individual factors affecting the mental health of older adults, some dimensions of SES at the household level have a lasting effect that serves as a resource or buffer against hardships in later years of life [20–22]. Moreover, resources such as income and assets accumulate over time; whereas, role-related deprivations could lead older adults to feel unfit in the family and ultimately to their cumulative disadvantages and increased psychological distress [22–24]. According to one study [25], well-being declines by increasing age and the situation is particularly worse for the oldest old segment and females tend to have a lesser well-being score as compared with males. Similarly, the lack of inheritance rights and property, and insufficient incomes and earnings expose older widows to deprivation and social isolation [26], resulting in a poor late-life mental health status.

Studies found a positive effect of co-residence with children on mental health and physical well-being [27]. Social networks, family dynamics, and both positive and negative aspects of these relationships are central to the well-being and functioning of older men and women [26]. At the same time, the study also reported that living in a multigenerational family without a spouse and having a lower household income were significantly associated with poor mental health in both men and women [28]. Evidence shows a linkage of perceived discrimination among older adults by their family members with their poor health including weak emotional states such as anxiety and depression [29–31]. Similarly, financial resources, better health, availability, and quality of social care are very high in urban areas [32]. Hence, differences were found between well-being in rural and urban older populations due to the socio-demographic factors, social resources, and income adequacy [32].

Various studies in India have analysed the risk factors such as lower levels of income, not working, not receiving any pension, not owning any asset, being a woman, and not having an adult child for care and support that are associated with mental health among older adults [33–36]. However, there is less on the association of particular SES indicators with psychological health and SWB. Among the ones that do so, very few specifically analyse the subjective income status and its association with mental health outcomes. In addition, self-perceived income sufficiency is recommended as a useful question in assessing health outcomes of vulnerable populations [37]. Since better psychological health and SWB are associated with positive health outcomes and increased longevity [38], the present study contributes to the literature on understanding the association of socioeconomic variables and self-perceived income status in particular, with mental health outcomes (psychological distress and SWB) among older adults (60 years and above) in India. The study hypothesized that:-

H_1_: There is a positive association of self-perceived income status of older adults with their psychological health and subjective well-being.

H_2_: Poor socioeconomic status among older adults is significantly associated with increased psychological distress and low subjective well-being.

## Methods

Data for this study is derived from the BKPAI (Building Knowledge Base on Population Ageing in India) which was conducted in 2011 [39]. The survey was carried out in seven major states (Himachal Pradesh, Punjab, West Bengal, Odisha, Maharashtra, Kerala and Tamil Nadu), that covered 9852 elders from 8329 older adult’s households both from rural and urban areas [39]. The states selected for the survey had higher percentage of the 60 + population compared to the national average [39]. The individual questionnaire covers on the socio-demographic profile, work history and benefit, income and assets, living arrangement, social activities, the health status of older adults & social security related questions [39].

### Sampling procedure

The BKPAI sample design entails a two-stage probability sampling. Where first villages were classified into different strata based on population size and the number of PSUs to be selected was determined in proportion to the population size of each stratum [39]. Using probability proportional to population size (PPS) technique, the primary sampling unit (PSUs) have been chosen, and within each selected PSU, older households were selected through systematic sampling. A similar procedure was applied in drawing samples from urban areas [39]. The final sample size for the analysis after removing missing cases and outliers was 9231 older adults aged 60 years and above.

### Outcome variable

The general health questionnaire, most common assessment of mental well-being is used as a measure of the common mental health problems/domains of depression, anxiety, somatic symptoms, and social withdrawal [40]. Further, SWB provides a meaningful and complementary measure of the health of older adults as it involves the subjective appraisals of their life in older age from their own perspective [41, 42]. The 12-item version of the General Health Questionnaire (GHQ-12) was used as a measure of mental health in the study. Psychological distress was having a scale of 0 to 12 based on experiencing stressful symptoms and was recoded as 0 “high” (representing 6 + scores) and 1 “low” (representing score 5 and less) [43, 44] (Cronbach's alpha: 0.90).

The 9-item Subjective Well-being Inventory was used to measure low subjective well-being. Subjective wellbeing inventory having a scale of 0 to 9 and was categorized as 0 “high” experiencing better experience (representing 6 + scores) and 1 “low” experiencing negative experience (representing score 5 and less) [45]. Twelve questions on psychological distress and nine questions on SWB were asked to assess the outcomes. All the questions on the outcome variables were asked on a Likert scale and were recoded to a dichotomy and used as per the previous literature [46]. The low SWB represents lower levels of subjective well-being among older adults (Cronbach's alpha: 0.93).

### Explanatory variables

Self-perceived income sufficiency was recoded as (no income, has income and fully sufficient, has income and partially sufficient and has income and not sufficient), working status (in last 1 year) was recoded as (never worked, currently working and retired) [47], receiving pension (no and yes), asset ownership was asked regarding homeownership, land ownership, jewellery ownership and other monetary savings and was recoded as (no and yes). Sex (men and women) and place of residence (rural and urban) were considered in the analysis. Co-residing with children was recoded as (no and yes). Age was recoded as ‘60–69 years, 70–79 years and 80 + years’, educational status was recoded as ‘no education, below five years, 6–10 years and 11 + years’ and marital status was recoded as ‘not in a marital union and currently in union’ [33].

Decision-making power was assessed through the question “who usually makes the following decisions: you alone or with your spouse, with your children, or with others?” on the following issues a. marriage of son/daughter. b. buying and selling of property c. buying other household items d. gifts to daughters, grandchildren, other relatives e. education of children, grandchildren f. arrangement of social and religious events (Cronbach alpha: 0.88). The variable decision making power was thus recoded as (no role, partial role, and absolute role). Community involvement was coded as (no and yes) [48]. Have someone to trust was coded as (no and yes) [34]. Experienced economic violence was recoded as (no and yes) [33, 49]. Chronic diseases were coded as (no and yes) [49]. Caste was categorized as Scheduled Castes, Scheduled Tribes, Other Backward Classes and others [50]. Religion was recoded as Hindus, Muslims, Sikhs and others, household wealth index was divided into five quintiles i.e. poorest, poorer, middle, richer and richest. The wealth index drawn based on the BKPAI survey is based on the following 30 assets and housing characteristics: household electrification; drinking water source; type of toilet facility; type of house; cooking fuel; house ownership; ownership of a bank or post-office account; and ownership of a mattress, a pressure cooker, a chair, a cot/bed, a table, an electric fan, a radio/transistor, a black and white television, a colour television, a sewing machine, a mobile telephone, any landline phone, a computer, internet facility; a refrigerator, a watch or clock, a bicycle, a motorcycle or scooter, an animal-drawn cart, a car, a water pump, a thresher, and a tractor. The range of index was from poorest to the richest i.e. ranging from lowest to the highest [39].

### Statistical analysis

Descriptive analysis along with bivariate analysis was employed to find the plausible association between psychological distress and low SWB with exposure and potential risk factors using the chi-square test. Apart from this, binary logistic regression analysis [51] was conducted to understand the relationship between psychological distress and low SWB and other risk factors. The software used was Stata 14 [52]. The significance level was set to be 5% (*p* < 0.05). Svyset command was used to control the analysis for complex survey design. Additionally, individual weights were used during the analysis to make the estimates nationally representative.

## Results

Table 1 represents the socio-economic and demographic profile of older adults in India. The sample represents the Indian older adult population. About 43% of older adults had no income whereas 7% has income but not sufficient to fulfil their basic needs. Nearly, 9% of older adults were retired from regular employment. Almost, 70% older adults had no pension and nearly, 18% of older adults had no asset ownership. About 53% of older adults were women and nearly 26% of older adults belong to rural areas. Nearly, 30% of older adults do not co-reside with their children. Eleven per cent of older adults belong to 80 and above age group. Nearly, 51% of older adults had no education and only 6% had 11 and above years of education. About, 40% of older adults were no in marital union during the survey period. Nearly, 70% of older adults had an absolute role in decision making in the household. About 20.5% and 17.3% of older adults had no community involvement and had no one to trust respectively. Almost, 5% of older adults reported that they suffered from some type of economic abuse after turning age 60. About 35.4% of older adults suffered from chronic diseases. About, 24% of older adults belonged to the poorest wealth quintile and 15% belong to richest wealth quintile households.

**Table 1:** Socio-economic and demographic profile of older adults interviewed in India

Background characteristics	Sample	Percentage
*Self-perceived income sufficiency*		
No income	3,967	43.0
Has income and fully sufficient	2,168	23.5
Has income and partially sufficient	2,433	26.4
Has income and not sufficient	663	7.2
*Working status (last one year)*		
Never worked	6,212	67.3
Currently working	2,223	24.1
Retired	796	8.6
*Receiving pension*		
No	6,447	69.8
Yes	2,784	30.2
*Asset ownership*		
No	1,630	17.7
Yes	7,601	82.3
*Sex*		
Male	4,372	47.4
Female	4,859	52.6
*Co-residing with children*		
No	2,738	29.7
Yes	6,493	70.3
*Age group (in years)*		
60–69	5,704	61.8
70–79	2,536	27.5
80 +	991	10.7
*Educational status*		
No education	4,684	50.7
Below 5 years	1,900	20.6
6 to 10 years	2,086	22.6
11 + years	562	6.1
*Marital status*		
Not in union	3,649	39.5
Currently in union	5,582	60.5
*Decision making power*		
No role	512	5.6
Partial role	2,218	24.0
Absolute role	6,501	70.4
*Community involvement*		
No	1896	20.5
Yes	7335	79.5
*Have someone to trust*		
No	1600	17.3
Yes	7631	82.7
*Experienced economic violence*		
No	8,781	95.1
Yes	450	4.9
*Chronic diseases*		
No	3268	35.4
Yes	5963	64.6
*Caste*		
Scheduled Castes	1,911	20.7
Scheduled Tribes	515	5.6
Other Backward Classes	3,364	36.4
Others	3,441	37.3
*Religion*		
Hindus	7,324	79.3
Muslims	651	7.1
Sikhs	870	9.4
Others	386	4.2
*Household wealth status*		
Poorest	2,169	23.5
Poorer	2,029	22.0
Middle	1,913	20.7
Richer	1,720	18.6
Richest	1,399	15.2
*Place of residence*		
Rural	6,827	74.0
Urban	2,404	26.0
*State*		
Himachal Pradesh	1,471	15.9
Punjab	1,279	13.9
West Bengal	1,128	12.2
Odisha	1,454	15.8
Maharashtra	1,229	13.3
Kerala	1,341	14.5
Tamil Nadu	1,330	14.4
*Total*	9,231	100.0

Percentage of older adults suffering from psychological distress and low SWB in India were presented in Table 2. About 23.4 and 26.7% of older adults had psychological distress and low SWB respectively.

**Table 2:** Percentage of older adults suffering from psychological distress and low SWB in India

Background characteristics	Psychological distress		Low subjective well-being	
	(%)	*p* < 0.05	(%)	*p* < 0.05
*Self-perceived income sufficiency*		*		*
No income	27.1		30.8	
Has income and fully sufficient	13.5		15.6	
Has income and partially sufficient	23.0		26.6	
Has income and not sufficient	35.4		39.4	
*Working status (last one year)*		*		*
Never worked	27.1		30.4	
Currently working	19.4		23.5	
Retired	6.2		7.0	
*Receiving pension*		*		*
No	23.9		27.6	
Yes	22.3		24.6	
*Asset ownership*		*		*
No	31.1		36.5	
Yes	21.8		24.6	
*Sex*		*		*
Male	21.1		23.9	
Female	25.5		29.3	
*Co-residing with children*		*		*
No	24.6		29.9	
Yes	22.9		25.4	
*Age group (in years)*		*		*
60–69	19.9		23.2	
70–79	27.0		30.0	
80 +	34.9		38.7	
*Educational status*		*		*
No education	30.6		35.5	
Below 5 years	22.3		24.0	
6 to 10 years	12.5		13.9	
11 + years	8.4		10.3	
*Marital status*		*		*
Not in union	28.5		32.9	
Currently in union	20.1		22.7	
*Decision making power*		*		*
No role	50.3		55.9	
Partial role	28.5		33.0	
Absolute role	19.6		22.3	
*Community involvement*		*		*
No	34.5		40.2	
Yes	20.5		23.5	
*Have someone to trust*		*		*
No	37.4		42.8	
Yes	20.5		23.3	
*Experienced economic violence*		*		*
No	22.5		25.8	
Yes	42.0		45.0	
*Chronic diseases*		*		*
No	20.0		23.3	
Yes	25.3		28.6	
*Caste*		*		*
Scheduled Castes	28.0		33.8	
Scheduled Tribes	32.4		35.1	
Other Backward Classes	25.7		27.8	
Others	17.3		20.5	
*Religion*		*		*
Hindus	25.7		28.5	
Muslims	23.1		29.9	
Sikhs	7.8		12.1	
Others	16.3		21.6	
*Household wealth status*		*		*
Poorest	37.1		47.3	
Poorer	29.6		32.4	
Middle	19.7		21.1	
Richer	14.7		14.7	
Richest	9.2		9.3	
*Place of residence*		*		*
Rural	25.1		28.3	
Urban	18.7		22.4	
*State*		*		*
Himachal Pradesh	17.0		15.1	
Punjab	7.2		11.4	
West Bengal	29.3		48.3	
Odisha	37.5		35.3	
Maharashtra	22.7		34.3	
Kerala	13.8		14.8	
Tamil Nadu	36.2		31.7	
*Total*	23.4		26.7	

^*^if *p* < 0.05 based on chi-square test

Older adults who had income but that was not sufficient for the fulfilment of basic needs had the highest prevalence of psychological distress (35.1%) and low SWB (39.4%). Older adults who never worked had the highest prevalence of psychological distress (27.1%) and low SWB (30.4%). Older adults who do not have pension had a higher prevalence of psychological distress (23.9%) and low SWB (27.6%). Those older adults who do not own any asset had a higher prevalence of psychological distress (31.1%) and low SWB (36.5%). Older women had a higher prevalence of psychological distress (25.5%) and low SWB (29.3%). Older adults who were not co-residing with their children had a higher prevalence of psychological distress (24.6%) and low SWB (29.9%). Older adults with age 80 years and above had a higher burden of psychological distress (34.9%) and low SWB (38.7%).

Older adults with no education had a higher prevalence of psychological distress (30.6%) and low SWB (35.5%). About 28.5% and 32.9% of older adults who were not in union had psychological distress and low SWB respectively. No role in household decision making was the risk factor for higher prevalence of psychological distress (50.3%) and low SWB (55.9%). The older adults who do not had any community involvement and no one to trust on had a higher prevalence of psychological distress and lower subjective well-being. Older adults who faced economic violence had a higher prevalence of psychological distress (42.0%) and low SWB (45.0%). Older adults who had a higher prevalence of chronic diseases had a higher prevalence of psychological distress (25.3%) and low subjective wellbeing (28.6%). About, 32.4 and 35.1% of older adults who belong to Scheduled Tribe had psychological distress and low SWB respectively. Older adults who belong to the poorest wealth quintile households had the highest prevalence of psychological distress (37.1%) and low SWB (47.3%). Rural residents had a higher prevalence of psychological distress (25.1%) and low SWB (28.3%). Older adults from Odisha had the highest prevalence of psychological distress (37.5%) and older adults from West Bengal had the highest prevalence of low SWB (48.3%).

**Fig. 1:**
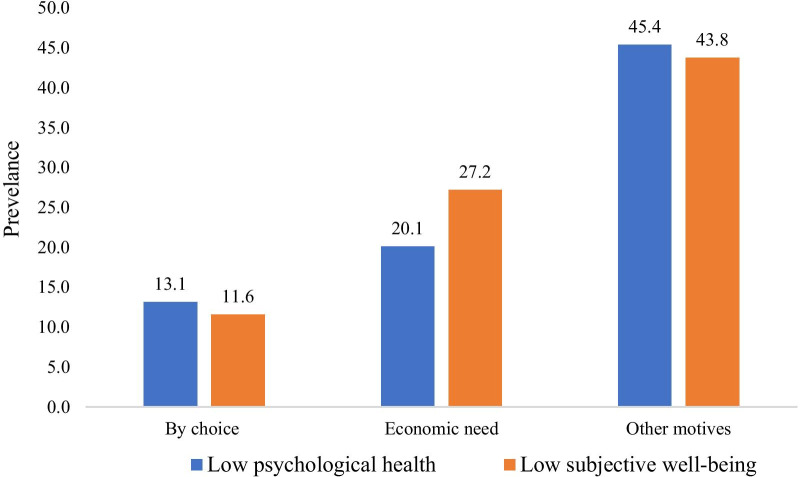
Prevalence of psychological distress and low SWB among older adults by their motivation of work

Figure 1 reveals the fact that older adults who worked by other motives includes involuntary works to meet the needs of their household had higher prevalence of psychological distress (45.4%) and low SWB (43.8%).

**Fig. 2:**
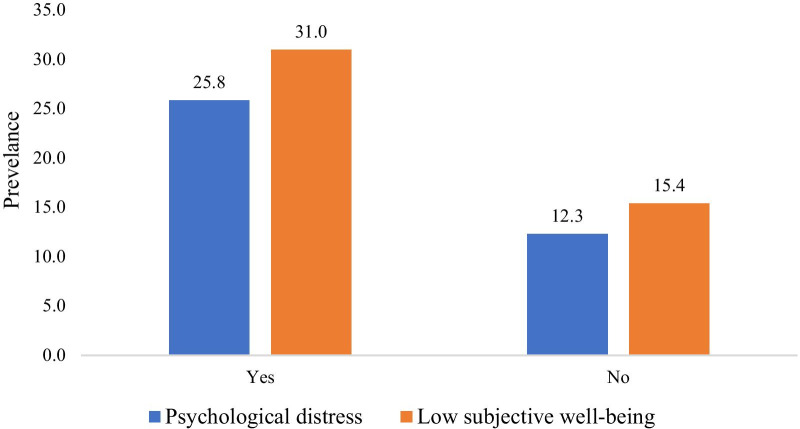
Prevalence of psychological distress and low SWB among older adults by mental or physical stress due to work

Figure 2 reveals that an older adult who experienced mental or physical stress due to work has a higher prevalence of psychological distress (25.8%) and low SWB (31.0%).

Table 3 represents logistic regression estimates for psychological distress and low SWB among older adults in India. Older adults with income perceived as partially sufficient to fulfil their basic needs had 2.23 times [OR: 2.23, CI: 1.75–2.84] and 1.96 times [OR: 1.96, CI: 1.55–2.47] significantly higher odds to suffer from psychological distress and low SWB respectively than older adults who had income perceived as sufficient. Older adults who never worked had 1.25 times [OR: 1.25, CI: 1.02–1.53] significantly higher odds to suffer from psychological distress than older adults who were currently working. Older adults with no asset ownership had 1.34 times [OR: 1.34, CI: 1.16–1.54] and 1.32 times [OR: 1.32 CI: 1.15–1.52] significantly higher odds to suffer from psychological distress and low SWB respectively than those who have assets.

**Table 3:** Logistic regression estimates for psychological distress and low SWB among older adults in India

Background characteristics	Psychological distress	Low subjective well-being
	OR (95% CI)	OR (95% CI)
*Self-perceived income sufficiency*		
No income	1.52*(1.27,1.82)	1.42*(1.2,1.69)
Has income and fully sufficient	Ref	Ref
Has income and partially sufficient	2.23*(1.75,2.84)	1.96*(1.55,2.47)
Has income and not sufficient	1.72*(1.36,2.17)	1.70*(1.36,2.13)
*Working status (last one year)*		
Never worked	1.25*(1.02,1.53)	1.18(0.97,1.43)
Currently working	Ref	Ref
Retired	0.52*(0.37,0.73)	0.52*(0.38,0.72)
*Receiving pension*		
No	0.76*(0.63,0.93)	0.78*(0.65,0.95)
Yes	Ref	Ref
*Asset ownership*		
No	1.34*(1.16,1.54)	1.32*(1.15,1.52)
Yes	Ref	Ref
*Sex*		
Male	Ref	Ref
Female	0.68*(0.59,0.79)	0.76*(0.66,0.87)
*Co-residing with children*		
No	1.05(0.93,1.20)	1.21*(1.07,1.37)
Yes	Ref	Ref
*Age group (in years)*		
60–69	Ref	Ref
70–79	1.34*(1.18,1.53)	1.37*(1.21,1.55)
80 +	1.81*(1.51,2.16)	1.82*(1.52,2.17)
*Educational status*		
No education	2.17*(1.55,3.02)	2.04*(1.49,2.81)
Below 5 years	1.59*(1.14,2.22)	1.5*(1.09,2.06)
6 to 10 years	0.98(0.71,1.36)	1.05(0.77,1.44)
11 + years	Ref	Ref
*Marital status*		
Not in union	1.16*(1.02,1.33)	1.14*(1.01,1.30)
Currently in union	Ref	Ref
*Decision making power*		
No role	1.79*(1.44,2.23)	2.14*(1.72,2.67)
Partial decision making	1.24*(1.09,1.40)	1.31*(1.16,1.48)
Absolute role	Ref	Ref
*Community involvement*		
No	1.44*(1.26,1.64)	1.51*(1.33,1.72)
Yes	Ref	Ref
*Have someone to trust*		
No	1.38*(1.20,1.59)	1.56*(1.36,1.80)
Yes	Ref	Ref
*Experienced economic violence*		
No	Ref	Ref
Yes	2.88*(2.26,3.67)	1.69*(1.33,2.15)
*Chronic diseases*		
No	Ref	Ref
Yes	1.83*(1.61,2.07)	1.68*(1.49,1.89)
*Caste*		
Scheduled Caste	1.21*(1.03,1.41)	1.22*(1.05,1.42)
Scheduled Tribe	0.93(0.72,1.19)	0.92(0.72,1.17)
Other Backward Class	0.98(0.84,1.15)	1.20*(1.04,1.39)
Others	Ref	Ref
*Religion*		
Hindu	Ref	Ref
Muslims	1.18(0.94,1.48)	1.14(0.92,1.42)
Sikh	0.99(0.67,1.46)	1.07(0.77,1.50)
Others	0.96(0.70,1.33)	1.08(0.8,1.45)
*Household wealth status*		
Poorest	1.56*(1.22,1.99)	3.01*(2.38,3.81)
Poorer	1.63*(1.30,2.04)	2.26*(1.82,2.81)
Middle	1.41*(1.14,1.76)	1.64*(1.33,2.03)
Richer	1.19(0.96,1.48)	1.39*(1.13,1.72)
Richest	Ref	Ref
*Place of residence*		
Rural	Ref	Ref
Urban	0.93(0.82,1.05)	1.11(0.99,1.25)
*State*		
Himachal Pradesh	Ref	Ref
Punjab	0.40*(0.29,0.57)	0.73(0.54,1.00)
West Bengal	1.83*(1.46,2.30)	4.16*(3.33,5.19)
Orissa	2.39*(1.92,2.97)	2.02*(1.61,2.52)
Maharashtra	1.12(0.89,1.41)	2.51*(2.02,3.13)
Kerala	0.83(0.64,1.07)	1.01(0.79,1.31)
Tamil Nadu	3.39*(2.68,4.29)	1.99*(1.57,2.52)

Ref: Reference category; CI: Confidence interval; OR: Odds Ratio; *if *p* < 0.05

Interestingly, different from the results from Table 2, in the adjusted model, women had a significantly lower likelihood to suffer from psychological distress [OR: 0.68, CI: 0.59–0.79] and low SWB [OR: 0.76, CI: 0.66–0.87] than their male counterparts. Older adults who do not co-reside with their children had a 21% [OR: 1.21, CI: 1.07–1.37] significantly higher likelihood to suffer from low SWB. Older adults who had no role in household decision making had 1.79 times [OR: 1.79, CI: 1.44–2.23] and 2.14 times [OR: 2.14, CI: 1.72–2.67] significantly higher odds to suffer from psychological distress and low SWB than older adults who had an absolute role in the household decision making. An older adults with no community involvement and no one to trust on had 1.44 times [OR: 1.44; CI: 1.26–1.64] and 1.38 times [OR: 1.38; CI: 1.20–1.59] significantly higher odds to suffer from psychological distress than their counterpart. Similar results were observed for low subjective well-being among older adults Economic violence proved to be fatal for psychological distress [OR: 2.88, CI: 2.26–3.67] and low SWB [OR: 1.69, CI: 1.33–2.15]. Older adults who suffered from chronic diseases had 1.83 times [OR: 1.83; CI: 1.61–2.07] and 1.68 times [OR: 1.68; CI: 1.49–1.89] significantly higher odds to suffer from psychological distress and low subjective wellbeing in reference to their counterpart. Additionally, advancing age, lack of education, not in marital union and poorest wealth status proved to be significant risk factors for psychological distress and low SWB among older adults. Older adults from Tamil Nadu had 3.39 times [OR: 3.39, CI: 2.68–4.29] significantly higher odds to suffer from psychological distress and older adults from West Bengal had 4.16 times [OR: 4.16, CI: 3.33–5.19] significantly higher odds to suffer from low SWB than older adults from Himachal Pradesh. Additional file 1: Tables S1 and S2 represents the segregated analysis for sex and place of residence. Both tables provide sensitivity analysis for psychological distress and low subjective well-being.

## Discussion

Even though there were several notable exceptions, most of the indicators of SES were strongly related to two of the mental health outcomes considered in the present study. Financial independence is important for older adults for keeping a quality life in old age. However, a large proportion of older individuals in our study who currently work for their economic need or by compulsion and not by choice had lower levels of mental health outcomes, indicating that the frustration from a job is a major risk factor among older Indian adults. The present study found that about 43% of older adults had no income source. The findings were somewhat relatable with previous studies. In Korea the poverty rates among older population rose from 29% in 1996 to 41% in 2014 [53]. Similarly in China it was found that in 2008 about 15% of older adults consumed at levels below the World Bank’s $1.25/day international poverty line [54]. Another study in China revealed that about 33% of older adults were identified as falling in poverty [55].

The present study found that the self-perceived income status is more associated with mental health conditions than any other single indicator of SES. Earlier studies have demonstrated that the financial strain in older adults may act as a stressor that would exacerbate other ongoing deterioration in their health outcomes [56]. Additionally, a recent study found that perceiving a lower income status is associated with lower life satisfaction and lower levels of happiness [57]. Again, own assets and accumulated wealth are the primary sources of support for older persons in Asian countries, whereas, older persons in the West rely heavily on public transfers [1]. Consistently, in our study, those who perceived income as sufficient to fulfil their basic needs and those who had asset ownership reported better mental health outcomes than their counterparts.

On the other hand, in developing countries like India, few people look forward to retirement, whereas the majority with their unmet life desires dreads it [58]. And due to the poor social security, older adults continuing to work beyond the retirement age is a norm in India [59]. Besides, working post-retirement is a positive factor in maintaining psychological well-being among older individuals [60]. In concordance with this, our study found that those who retired from work have better mental health and SWB. A striking gender difference in reporting poor mental health and SWB was also found in our study. Men suffered more psychological distress than women. It might be due to the burden of domestic chores and their lack of social networks and support after they lose their job or spouse [61]. Interestingly, it was also found that older adults who are currently married experience better psychological and subjective wellbeing.

Furthermore, despite the efforts of government interventions, the coverage of the Indian old-age pension system has remained low due to the discretionary or voluntary nature of the schemes [62]. A recent study observed that the pension receipt directly affects the well-being of retired older adults with low economic status [63]. Further, the pension receipt is found to be associated with increased household expenditure, indicating that most of the income from pension received is used for either improving the health or educational outcomes of other family members [64]. A recent study also observed that though the households spent most of the old-age pension income on improving overall family welfare, it reduced the work participation of older adults substantially [65]. In line with this, older adults in our study who received pension reported poor mental health outcomes.

The studies on the association of education with health outcomes in later years suggest that older people with higher levels of education may have a better understanding of their ageing process and reap the benefit of quality care services and better health [66]. Similarly, the literates in our study also reported higher levels of mental and SWB. Furthermore, older people in India traditionally have lived with their children or grandchildren. Such living arrangements are found to be mutually beneficial with older parents providing childcare and other forms of support in domestic work and receiving economic support and care in return [67]. The study suggests that in cultures in which intergenerational ties have higher value, co-residing with children is positively associated with the mental health of the older population [28]. As the evidence suggests, the difference between the rich and the poor in any population extends far beyond money alone. For instance, previous studies observed that the quality relationships rather than the number of family ties were associated with feelings of well-being [68, 69]. In countries where care and support toward older parents is a social norm, it is found that co-residence was associated with a low prevalence of depressive symptoms [70, 71]. When their spouses die, men lose much of the support and care that wives provide, such as emotional support and the maintenance of social contact with children and others [72]. Consistently, our study found that current marital status and living with an adult child were positively associated with better psychological health and higher SWB.

The results show that factors that were significantly associated with the outcomes were primarily related to the older adults themselves. In fact, few other factors were also found to be related to psychological and SWB in old age. They include older adults’ importance in the decision making role in the household, experiencing economic abuse within the family, household wealth status, etc. On the other hand, old age is seen as a time of major losses of social roles and experiences of deteriorating both the quality and quantity of relationships [73]. Partial or absolute role in household decision making in this study is found positively associated with mental health outcomes among older adults. Further, the increased burden of low social status makes people feel disrespected and older people are subjected to several types of domestic abuse [74]. And economic abuse is found as a negative predictor of overall psychological disorders among older adults [75]. Consistently, the results of the current study show that those older adults who reported economic abuse have low psychological and subjective health outcomes.

The positive association of increasing age with poor mental health is in parallel with findings in India showing the increased age as a major predictor of late-life depression [76]. Also, considering the poor well-being score among male older population, the grim scenario of gender disparity in later life is evident, thus throwing light on the male disadvantage in mental health and the cultural paradox that persist in India [25], and more investigation is warranted in this regard. Furthermore, our analysis is also in consistence with the finding that psychological health may also be affected by different factors for older individuals who are from households in which poverty is more common than for individuals from households with more assets and higher incomes [77]. The results show that older adults from the richest wealth quintile had better mental health status compared to all other quintiles. The case is similar with regard to the older adults’ place of residence too. Although the multivariate analyses showed no significance, the bivariate results noticed the poor mental status of older adults residing in rural areas of the country, indicating that rural location as a risk factor for ill-being in older ages [2, 78].

The study poses several limitations such as the shared response biases that can occur when both outcome measures of mental health status and well-being are based on self-reports. Another limitation is that the data is cross-sectional in design and we are restricted in addressing causality between socioeconomic statuses and outcome variables. Moreover, though the study focuses on many aspects of socioeconomic and familial factors that affect the well-being of older adults, the findings are limited by imprecise measures.

## Conclusions

The study found that the illiterate, older women, older individuals with low levels of perceived income sufficiency, not working, not owning asset/home, not living with their children, experiencing economic abuse, and having no role in household decision making were all at increased risk for psychological distress and low SWB. In this regard, the study highlights that the existing social security system and care services in the country is inadequate to meet the multifaceted need of the growing older population.

By focusing on four target areas such as income support, education, family-oriented initiatives, and local or regional policies, the current framework for assessing the mental health among older adults in India can be modified. A move towards a guaranteed pension for eligible older individuals, by which they do not have to remain as a financial burden on their children may reduce their self-perceived economic distress and result in higher levels of wellbeing in older ages. Also, strategies to address socioeconomic disadvantages and gender differentials related to mental health status among older population are urgently needed.

## Supplementary Information

**Additional file 1. Table S1**. Logistic regression analysis for psychological distress and low subjective well-being among older male and female (60 years and above) in India.** Table S2**. Logistic regression analysis for psychological distress and low subjective well-being among older adults from rural and urban place of residence in India.

## Data Availability

The data that support the findings of this study are available from [director@isec.ac.in or india.office@unfpa.org] but restrictions apply to the availability of these data, which were used under license for the current study, and so are not publicly available. Data are however available from the authors upon reasonable request and with permission of [director@isec.ac.in or india.office@unfpa.org]**.**

## Abbreviations

SRH: Self-rated health
ADL: Activities of daily living
IADL: Instrumental activities of daily living
OR: Odds ratio
CI: Confidence Interval
BKPAI: Building a Knowledge Base on Population Aging in India
SWB: Subjective well-being
