# Supplementary material for: Association of self-perceived income status with psychological distress and subjective well-being: a cross-sectional study among older adults in India
Source: BMC Psychol. 2021 May 18;9:82. doi: 10.1186/s40359-021-00588-5 (PMC8130272; doi:10.1186/s40359-021-00588-5)
Supplement: Supplementary file 1 — Additional file 1. Table S1. Logistic regression analysis for psychological distress and low subjective well-being among older male and female (60 years and above) in India. Table S2. Logistic regression analysis for psychological distress and low subjective well-being among older adults from rural and urban place of residence in India. [file 40359_2021_588_MOESM1_ESM.docx]

| **Table-S1. Logistic regression analysis for psychological distress and low subjective well-being among older male and female (60 years and above) in India** | | | | |
| --- | --- | --- | --- | --- |
| **Background characteristics** | **Psychological distress** | | **Low subjective well-being** | |
|  | **Male** | **Female** | **Male** | **Female** |
|  | **OR (95% CI)** | **OR (95% CI)** | **OR (95% CI)** | **OR (95% CI)** |
| **Self-perceived income sufficiency** |  |  |  |  |
| No income at all | 1.59*(1.25,2.01) | 1.31(0.99,1.72) | 1.54*(1.23,1.93) | 1.17(0.9,1.52) |
| Has income and fully sufficient | Ref. | Ref. | Ref. | Ref. |
| Has income and partially sufficient | 3.24*(2.29,4.59) | 1.41(0.99,1.99) | 2.44*(1.73,3.44) | 1.4*(1.01,1.96) |
| Has income and not sufficient | 2.02*(1.47,2.77) | 1.41(0.97,2.04) | 1.85*(1.36,2.53) | 1.47*(1.03,2.08) |
| **Working status (last one year)** |  |  |  |  |
| Never worked | 1.25(0.96,1.63) | 1.05(0.76,1.46) | 1.5*(1.15,1.94) | 0.69*(0.5,0.93) |
| Currently working | Ref. | Ref. | Ref. | Ref. |
| Retired | 0.54*(0.37,0.8) | 0.55(0.22,1.36) | 0.58*(0.39,0.85) | 0.72(0.35,1.51) |
| **Receiving pension** |  |  |  |  |
| No | 0.82(0.63,1.07) | 0.74(0.54,1.03) | 0.78(0.6,1.02) | 0.8(0.59,1.09) |
| Yes | Ref. | Ref. | Ref. | Ref. |
| **Asset ownership** |  |  |  |  |
| No | 1.31(0.98,1.75) | 1.47*(1.24,1.74) | 1.49*(1.12,1.97) | 1.32*(1.12,1.56) |
| Yes | Ref. | Ref. | Ref. | Ref. |
| **Co-residing with children** |  |  |  |  |
| No | 1.04(0.85,1.26) | 1(0.83,1.19) | 1.21(1,1.46) | 1.09(0.92,1.29) |
| Yes | Ref. | Ref. | Ref. | Ref. |
| **Age group (in years)** |  |  |  |  |
| 60-69 | Ref. | Ref. | Ref. | Ref. |
| 70-79 | 1.09(0.89,1.33) | 1.41*(1.19,1.67) | 1.06(0.87,1.29) | 1.5*(1.27,1.77) |
| 80+ | 1.47*(1.11,1.95) | 1.68*(1.32,2.14) | 1.36*(1.02,1.79) | 1.78*(1.4,2.27) |
| **Educational status** |  |  |  |  |
| No education | 1.98*(1.31,3) | 2.17*(1.16,4.09) | 1.98*(1.31,2.99) | 1.86*(1.07,3.22) |
| Below 5 years | 1.58*(1.04,2.39) | 1.54(0.82,2.9) | 1.73*(1.15,2.6) | 1.21(0.69,2.1) |
| 6 to 10 years | 1.07(0.72,1.58) | 0.9(0.47,1.71) | 1.22(0.83,1.8) | 0.89(0.51,1.55) |
| 11+ years | Ref. | Ref. | Ref. | Ref. |
| **Marital status** |  |  |  |  |
| Not in union | 1(0.8,1.25) | 1.13(0.94,1.36) | 0.97(0.78,1.21) | 1.07(0.91,1.28) |
| Currently in union | Ref. | Ref. | Ref. | Ref. |
| **Decision making power** |  |  |  |  |
| No role | 1.73*(1.08,2.75) | 1.54*(1.19,1.99) | 1.79*(1.12,2.87) | 1.98*(1.53,2.56) |
| Partial decision making | 1.2(0.99,1.47) | 1.28*(1.08,1.51) | 1.18(0.97,1.44) | 1.44*(1.23,1.7) |
| Absolute role | Ref. | Ref. | Ref. | Ref. |
| **Community involvement** |  |  |  |  |
| No | 1.47*(1.18,1.83) | 1.42*(1.2,1.68) | 1.69*(1.37,2.09) | 1.41*(1.2,1.66) |
| Yes | Ref. | Ref. | Ref. | Ref. |
| **Have someone to trust** |  |  |  |  |
| No | 1.36*(1.09,1.7) | 1.39*(1.15,1.67) | 1.66*(1.34,2.07) | 1.49*(1.24,1.78) |
| Yes | Ref. | Ref. | Ref. | Ref. |
| **Experienced economic violence** |  |  |  |  |
| No | 2.19*(1.49,3.22) | 2.98*(2.16,4.11) | 1.05(0.7,1.56) | 2*(1.46,2.75) |
| Yes | Ref. | Ref. | Ref. | Ref. |
| **Chronic diseases** |  |  |  |  |
| No | Ref. | Ref. | Ref. | Ref. |
| Yes | 1.64*(1.36,1.98) | 2.03*(1.71,2.41) | 1.63*(1.36,1.96) | 1.70*(1.45,2) |
| **Caste** |  |  |  |  |
| Scheduled Caste | 1.2(0.94,1.53) | 1.17(0.95,1.45) | 1.33*(1.05,1.68) | 1.09(0.88,1.34) |
| Scheduled Tribe | 1.11(0.76,1.62) | 0.96(0.69,1.35) | 1.11(0.77,1.61) | 0.92(0.66,1.29) |
| Other Backward Class | 1.13(0.89,1.44) | 0.9(0.73,1.11) | 1.26*(1,1.59) | 1.2(0.98,1.46) |
| Others | Ref. | Ref. | Ref. | Ref. |
| **Religion** |  |  |  |  |
| Hindu | Ref. | Ref. | Ref. | Ref. |
| Muslims | 1.04(0.72,1.52) | 1.2(0.89,1.6) | 1.14(0.81,1.61) | 1.13(0.84,1.5) |
| Sikh | 1.27(0.71,2.27) | 0.84(0.49,1.41) | 0.99(0.57,1.71) | 1.18(0.77,1.81) |
| Others | 1.05(0.61,1.8) | 0.87(0.58,1.31) | 1.03(0.63,1.69) | 1.11(0.75,1.63) |
| **Household wealth status** |  |  |  |  |
| Poorest | 1.62*(1.09,2.42) | 1.53*(1.11,2.12) | 3.53*(2.38,5.23) | 2.71*(1.98,3.7) |
| Poorer | 1.69*(1.17,2.44) | 1.62*(1.22,2.17) | 2.81*(1.94,4.05) | 1.99*(1.51,2.63) |
| Middle | 1.7*(1.2,2.41) | 1.29(0.97,1.71) | 2.15*(1.51,3.05) | 1.49*(1.13,1.95) |
| Richer | 1.36(0.96,1.93) | 1.17(0.88,1.56) | 1.74*(1.23,2.48) | 1.33*(1.01,1.74) |
| Richest | Ref. | Ref. | Ref. | Ref. |
| **Place of residence** |  |  |  |  |
| Rural | Ref. | Ref. | Ref. | Ref. |
| Urban | 0.9(0.74,1.09) | 0.99(0.84,1.16) | 1.13(0.93,1.36) | 1.16(0.99,1.36) |
| **State** |  |  |  |  |
| Himachal Pradesh | Ref. | Ref. | Ref. | Ref. |
| Punjab | 0.38*(0.22,0.65) | 0.36*(0.22,0.56) | 0.53*(0.32,0.88) | 0.81(0.54,1.22) |
| West Bengal | 2*(1.41,2.84) | 1.62*(1.19,2.2) | 3.69*(2.62,5.18) | 4.56*(3.37,6.17) |
| Orissa | 2.33*(1.66,3.27) | 2.42*(1.79,3.27) | 1.85*(1.31,2.61) | 1.94*(1.43,2.64) |
| Maharashtra | 1.04(0.73,1.49) | 1.01(0.74,1.39) | 1.93*(1.37,2.72) | 2.59*(1.92,3.5) |
| Kerala | 0.45*(0.29,0.71) | 1.02(0.73,1.44) | 0.67(0.44,1.01) | 1.14(0.81,1.59) |
| Tamil Nadu | 3.37*(2.34,4.86) | 3.38*(2.43,4.68) | 1.8*(1.24,2.62) | 1.84*(1.33,2.56) |
| Ref: Reference category; CI: Confidence interval; OR: Odds Ratio | | |  |  |

| **Table-S2. Logistic regression analysis for psychological distress and low subjective well-being among older adults from rural and urban place of residence in India** | | | | |
| --- | --- | --- | --- | --- |
| **Background characteristics** | **Low psychological health** | | **Low subjective well-being** | |
|  | **Rural** | **Urban** | **Rural** | **Urban** |
|  | **OR (95% CI)** | **OR (95% CI)** | **OR (95% CI)** | **OR (95% CI)** |
| **Income sufficient to fulfil basic need?** |  |  |  |  |
| No income at all | 1.25(0.99,1.58) | 1.9*(1.42,2.53) | 1.13(0.9,1.41) | 1.74*(1.33,2.27) |
| Has income and fully sufficient | Ref. | Ref. | Ref. | Ref. |
| Has income and partially sufficient | 2.23*(1.63,3.05) | 1.94*(1.3,2.88) | 1.65*(1.21,2.25) | 2.12*(1.46,3.06) |
| Has income and not sufficient | 1.36(1,1.85) | 2.15*(1.47,3.15) | 1.25(0.92,1.69) | 2.25*(1.57,3.23) |
| **Working status (last one year)** |  |  |  |  |
| Never worked | 1.37*(1.06,1.78) | 0.95(0.68,1.34) | 1.37*(1.06,1.76) | 0.78(0.57,1.08) |
| Currently working | Ref. | Ref. | Ref. | Ref. |
| Retired | 0.67(0.39,1.15) | 0.46*(0.29,0.74) | 0.71(0.41,1.23) | 0.42*(0.27,0.66) |
| **Receiving pension** |  |  |  |  |
| No | 0.9(0.69,1.16) | 0.66*(0.47,0.92) | 0.85(0.66,1.09) | 0.74(0.54,1.01) |
| Yes | Ref. | Ref. | Ref. | Ref. |
| **Asset ownership** |  |  |  |  |
| No | 1.22*(1,1.48) | 1.64*(1.32,2.03) | 1.3*(1.07,1.58) | 1.38*(1.13,1.7) |
| Yes | Ref. | Ref. | Ref. | Ref. |
| **Sex** |  |  |  |  |
| Male | Ref. | Ref. | Ref. | Ref. |
| Female | 0.71*(0.59,0.86) | 0.64*(0.51,0.8) | 0.75*(0.63,0.91) | 0.79*(0.64,0.98) |
| **Co-residing with children** |  |  |  |  |
| No | 1.02(0.87,1.21) | 0.98(0.79,1.21) | 1.13(0.97,1.33) | 1.16(0.95,1.41) |
| Yes | Ref. | Ref. | Ref. | Ref. |
| **Age group (in years)** |  |  |  |  |
| 60-69 | Ref. | Ref. | Ref. | Ref. |
| 70-79 | 1.13(0.96,1.34) | 1.45*(1.18,1.78) | 1.15(0.97,1.36) | 1.54*(1.27,1.86) |
| 80+ | 1.56*(1.22,1.98) | 1.62*(1.22,2.15) | 1.64*(1.28,2.08) | 1.63*(1.23,2.14) |
| **Educational status** |  |  |  |  |
| No education | 2.79*(1.47,5.3) | 1.92*(1.27,2.9) | 2.09*(1.16,3.74) | 1.81*(1.22,2.69) |
| Below 5 years | 1.82(0.96,3.46) | 1.69*(1.12,2.55) | 1.27(0.71,2.29) | 1.76*(1.19,2.6) |
| 6 to 10 years | 1.43(0.75,2.7) | 0.89(0.6,1.31) | 0.99(0.55,1.77) | 1.16(0.8,1.68) |
| 11+ years | Ref. | Ref. | Ref. | Ref. |
| **Marital status** |  |  |  |  |
| Not in union | 1.04(0.87,1.25) | 1.19(0.97,1.48) | 0.98(0.82,1.16) | 1.21(1,1.48) |
| Currently in union | Ref. | Ref. | Ref. | Ref. |
| **Decision making power** |  |  |  |  |
| No role | 1.46*(1.11,1.94) | 2.04*(1.4,2.97) | 1.77*(1.33,2.36) | 1.87*(1.3,2.71) |
| Partial decision making | 1.29*(1.09,1.52) | 1.24*(1.02,1.51) | 1.37*(1.16,1.62) | 1.31*(1.09,1.57) |
| Absolute role | Ref. | Ref. | Ref. | Ref. |
| **Community involvement** |  |  |  |  |
| No | 1.39*(1.17,1.65) | 1.59*(1.28,1.98) | 1.46*(1.23,1.73) | 1.58*(1.29,1.94) |
| Yes | Ref. | Ref. | Ref. | Ref. |
| **Trust over someone** |  |  |  |  |
| No | 1.29*(1.07,1.54) | 1.68*(1.33,2.11) | 1.62*(1.35,1.94) | 1.49*(1.19,1.87) |
| Yes | Ref. | Ref. | Ref. | Ref. |
| **Economic violence** |  |  |  |  |
| No | Ref. | Ref. | Ref. | Ref. |
| Yes | 2.51*(1.85,3.42) | 3.19*(2.12,4.78) | 1.59*(1.17,2.16) | 1.58*(1.06,2.36) |
| **Chronic diseases** |  |  |  |  |
| No | Ref. | Ref. | Ref. | Ref. |
| Yes | 1.73*(1.47,2.03) | 1.9*(1.55,2.32) | 1.44*(1.23,1.69) | 2.03*(1.69,2.46) |
| **Caste** |  |  |  |  |
| Scheduled Caste | 1.16(0.94,1.43) | 1.23(0.96,1.59) | 1.1(0.9,1.36) | 1.33*(1.05,1.69) |
| Scheduled Tribe | 0.98(0.73,1.33) | 0.96(0.58,1.59) | 1.06(0.78,1.42) | 0.88(0.54,1.43) |
| Other Backward Class | 0.95(0.77,1.18) | 1.01(0.79,1.29) | 1.22(0.99,1.5) | 1.19(0.95,1.49) |
| Others | Ref. | Ref. | Ref. | Ref. |
| **Religion** |  |  |  |  |
| Hindu | Ref. | Ref. | Ref. | Ref. |
| Muslims | 1.34(0.99,1.83) | 0.91(0.64,1.29) | 1.46*(1.07,1.99) | 0.85(0.62,1.18) |
| Sikh | 1.15(0.65,2.04) | 1.14(0.66,1.97) | 1.04(0.64,1.7) | 1.23(0.75,2.02) |
| Others | 1.06(0.66,1.71) | 0.86(0.55,1.34) | 1.35(0.85,2.12) | 0.88(0.58,1.32) |
| **Household wealth status** |  |  |  |  |
| Poorest | 1.37(0.93,2.01) | 1.94*(1.34,2.81) | 3.31*(2.24,4.89) | 2.69*(1.89,3.82) |
| Poorer | 1.56*(1.08,2.25) | 1.61*(1.18,2.2) | 2.44*(1.68,3.54) | 2.48*(1.85,3.32) |
| Middle | 1.43(0.99,2.06) | 1.42*(1.06,1.89) | 1.89*(1.3,2.75) | 1.67*(1.28,2.19) |
| Richer | 1.25(0.86,1.84) | 1.2(0.91,1.58) | 1.4(0.94,2.07) | 1.55*(1.2,2) |
| Richest | Ref. | Ref. | Ref. | Ref. |
| **State** |  |  |  |  |
| Himachal Pradesh | Ref. | Ref. | Ref. | Ref. |
| Punjab | 0.27*(0.16,0.47) | 0.45*(0.28,0.72) | 0.64(0.4,1.03) | 0.72(0.46,1.12) |
| West Bengal | 2.07*(1.53,2.82) | 1.59*(1.1,2.28) | 4.36*(3.22,5.92) | 3.96*(2.8,5.61) |
| Orissa | 2.98*(2.21,4.01) | 1.76*(1.23,2.51) | 1.86*(1.37,2.52) | 1.94*(1.36,2.78) |
| Maharashtra | 1.1(0.81,1.48) | 1.03(0.71,1.5) | 2.01*(1.49,2.69) | 3*(2.11,4.27) |
| Kerala | 0.79(0.56,1.11) | 0.76(0.5,1.16) | 0.94(0.67,1.32) | 1.01(0.67,1.51) |
| Tamil Nadu | 3.5*(2.55,4.79) | 4*(2.71,5.88) | 1.97*(1.43,2.71) | 1.91*(1.3,2.82) |
| Ref: Reference category; CI: Confidence interval; OR: Odds Ratio | | |  |  |
